# Supplementary material for: Cleavage of mRNAs by a minority of pachytene piRNAs improves sperm fitness
Source: Nature. 2026 Feb 4;652(8109):508–16. doi: 10.1038/s41586-026-10102-9 (PMC13061629; doi:10.1038/s41586-026-10102-9)
Supplement: Supplementary file 1 — Supplementary Discussion. [file 41586_2026_10102_MOESM1_ESM.docx]

**SUPPLEMENTARY INFORMATION**

**Cleavage of mRNAs by a minority of pachytene piRNAs improves sperm fitness**

Katharine Cecchini, Mina Zamani, Nandagopal Ajaykumar, Joel Vega-Badillo, Ayca Bagci, Shannon Bailey, Phillip D. Zamore & Ildar Gainetdinov

**SUPPLEMENTARY DISCUSSION**

**Changes in transcript abundance in *pi6^−/−^*, *pi9^−/−^*, and *pi17^−/−^* mutants that are not explained by piRNA-guided cleavage**

For some of the mRNAs whose derepression in *pi6^−/−^*, *pi9^−/−^*, and *pi17^−/−^* mutants was not explained by piRNA-guided cleavage, we hypothesize that their low abundance prevented detection of their cleavage products. We note that, for most of these, we found 5′-monophosphate-bearing RNAs in C57BL/6 that were reduced by >8-fold in piRNA mutants, but no piRNAs were identified that met the piRNA:target pairing requirements used in this study. It is possible that our current piRNA targeting rules are incomplete and *pi6*, *pi9*, or *pi17* piRNAs guide cleavage of these transcripts via pairing modes not yet identified.

**Pachytene piRNA-producing loci are dispensable for germ cell granule formation**

Several piRNA pathway proteins assemble into germline-specific, electron-dense structures called intermitochondrial cement in primary spermatocytes and chromatoid bodies in haploid spermatids^68‑70^. For example, the PIWI protein MIWI, bound to pachytene piRNAs, comprises a sixth of the proteins in chromatoid bodies^70^, and both intermitochondrial cement and chromatoid bodies are lost in *Miwi^−/−^* mutants (ref. ^71^; Extended Data Fig. 14). In contrast, loss of ~13% of pachytene piRNAs in *pi2^−/−^pi17^−/−^* or *pi9^−/−^pi17^−/−^* males had no detectable effect on germ cell granule morphology. The number of mitochondria and the abundance of intermitochondrial cement was essentially identical in primary spermatocytes from all analyzed genotypes: control C57BL/6, double-mutant *pi2^−/−^pi17^−/−^*, which are fertile, and *pi9^−/−^pi17^−/−^* males, which are sterile (Extended Data Fig. 14). Chromatoid body morphology in round spermatids from C57BL/6, *pi2^−/−^pi17^−/−^*, and *pi9^−/−^pi17^−/−^* was similarly indistinguishable (Extended Data Fig. 14). These findings demonstrate that both intermitochondrial cement and chromatoid bodies are intact in pachytene piRNA mutants, irrespective of their fertility.

**Pachytene piRNA-directed cleavage rarely influences the abundance of targets**

Pachytene piRNA-directed cleavage rarely changes the steady-state levels of target RNAs, likely because of the low efficiency of slicing by most pachytene piRNAs and the high transcription rates of their targets. We hypothesize that pachytene piRNAs that change the steady-state levels of targets are underrepresented because such piRNAs are typically deleterious and removed by negative selection.

An additional explanation for why pachytene piRNAs frequently target highly-transcribed genes is that deleterious piRNAs can survive if the population includes males with pre-existing, highly active promoter variants. In this scenario, piRNA-guided cleavage would select for target variants producing sufficient RNA to overcome the piRNA-guided target cleavage. Thus, the highly expressed promoter allele would provide a selectable advantage in the presence of the piRNA, and, over evolutionary time, the weaker promoter alleles would decrease in the population.
