# Supplementary material for: Cleavage of mRNAs by a minority of pachytene piRNAs improves sperm fitness
Source: Nature. 2026 Feb 4;652(8109):508–16. doi: 10.1038/s41586-026-10102-9 (PMC13061629; doi:10.1038/s41586-026-10102-9)
Supplement: Supplementary file 3 — Uncropped gel source data. Density of 5′ ends of RFP reads. Gene ontology terms enriched among mRNAs with changed translational efficiency. RFP-seq and RNA-seq data for Tbpl1, Spesp1 and Cnot4 mRNA. Change in mRNA translational efficiency in pachytene piRNA mutants. FACS gating strategy to purify mouse primary germ cells. [file 41586_2026_10102_MOESM3_ESM.pdf]

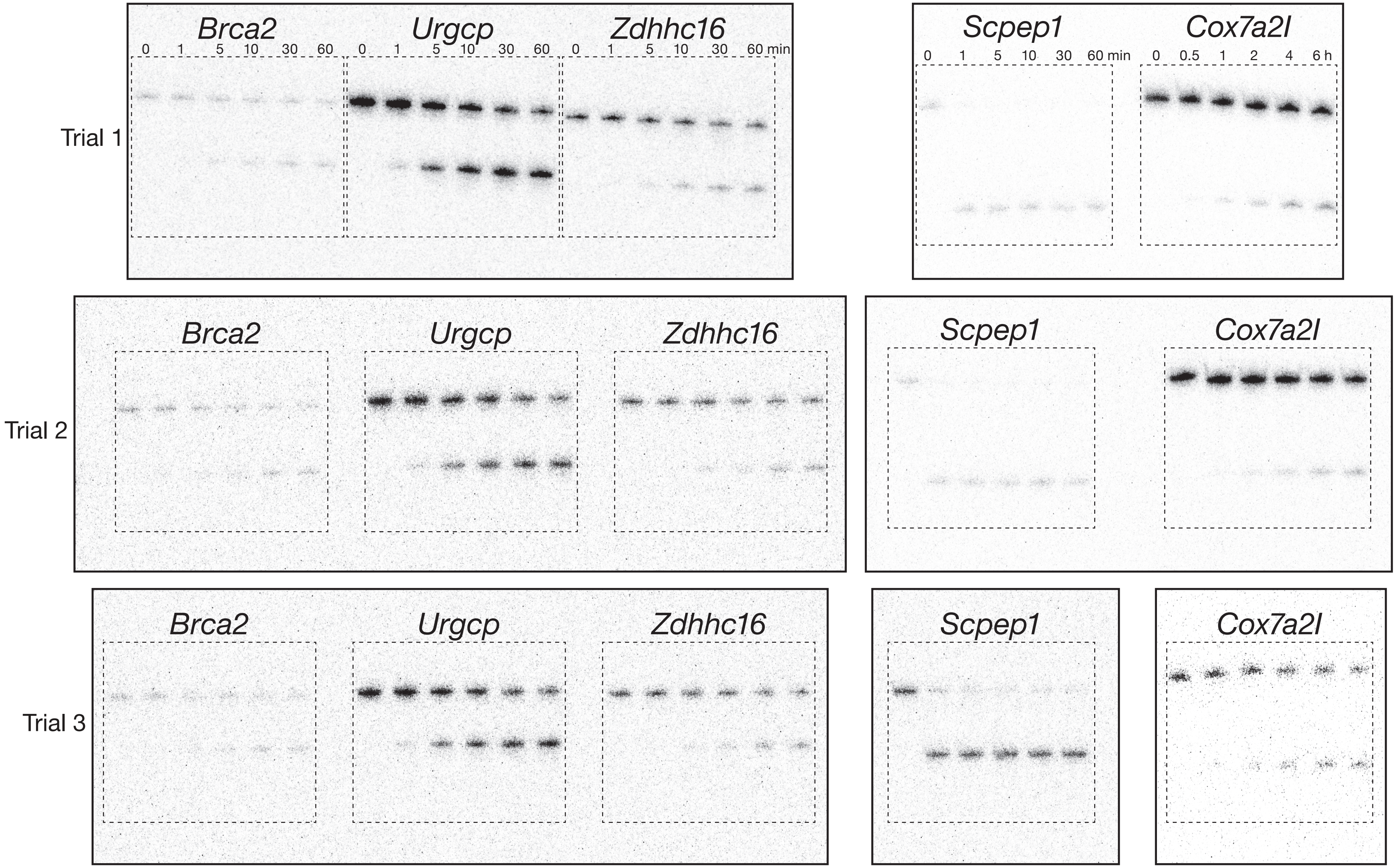

Supplementary Figure 1. Uncropped gel source data for Extended Data Fig. 3

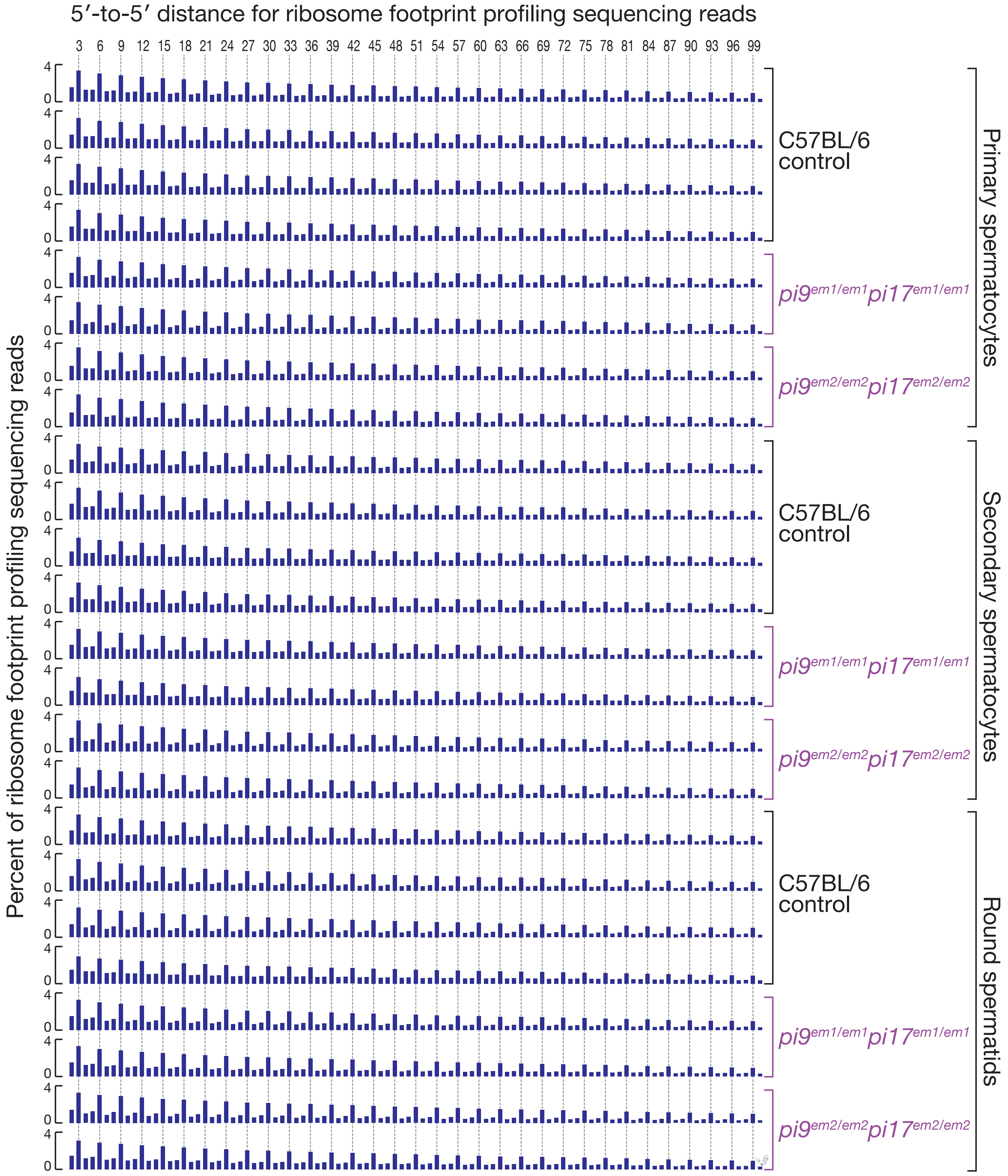

Supplementary Figure 2. Density of 5′ ends of RFP reads exhibit 3-nt periodicity.
Individual biological replicates for C57BL/6 controls (n = 4) and $pi9^{-/-}pi17^{-/-}$ (n = 4) are shown.

Comparison	Translational efficiency	Gene Ontology term (biological process)	Genes observed	Genes expected	Enrichment	Adjusted *p*-value
Round spermatids vs **secondary spermatocytes**	Translational efficiency **increases ≥2-fold** (89 genes)	acrosome assembly	4	0.18	22.8	1.7×10^{-2}
		binding of sperm to zona pellucida	4	0.20	20.0	2.8×10^{-2}
		flagellated sperm motility	13	0.86	15.1	6.3×10^{-9}
		male gonad development	6	0.73	8.3	4.8×10^{-2}
	Translational efficiency **decreases ≥1.5-fold** (45 genes)	microtubule cytoskeleton organization	9	1.23	7.3	4.8×10^{-2}

Comparison	Translational efficiency	Gene Ontology term (biological process)	Genes observed	Genes expected	Enrichment	Adjusted *p*-value
Round spermatids vs **primary spermatocytes**	Translational efficiency **increases ≥3-fold** (104 genes)	sperm mitochondrial sheath assembly	3	0.03	90.0	2.8×10^{-3}
		binding of sperm to zona pellucida	4	0.20	19.5	3.3×10^{-2}
		single fertilization	9	0.87	10.3	2.4×10^{-4}
	Translational efficiency **decreases ≥1.5-fold** (114 genes)	coding region determinant mRNA stabilization	3	0.06	51.8	1.2×10^{-2}
		IRES-dependent viral translational initiation	3	0.06	51.8	1.2×10^{-2}
		deadenylation-dependent decay	3	0.06	47.5	1.4×10^{-2}
		positive regulation of cytoplasmic translation	3	0.08	35.6	2.5×10^{-2}
		positive regulation of DNA-templated transcription	5	0.39	12.7	1.9×10^{-2}
		regulation of mRNA splicing, via spliceosome	7	0.65	10.8	3.1×10^{-3}
		nuclear export	6	0.7	8.6	2.5×10^{-2}
		RNA transport	6	0.76	7.9	3.1×10^{-2}
		circadian rhythm	7	0.93	7.6	1.7×10^{-2}
		protein localization to nucleus	7	1.11	6.3	3.1×10^{-2}
		spermatid development	9	1.67	5.4	1.8×10^{-2}
		mRNA processing	12	2.39	5.0	3.7×10^{-3}
		RNA splicing	9	1.93	4.7	3.4×10^{-2}
		chromosome organization	10	2.24	4.5	2.6×10^{-2}
		intracellular protein transport	12	2.81	4.3	1.3×10^{-2}
		regulation of DNA metabolic process	11	2.66	4.1	2.5×10^{-2}
		microtubule-based process	15	4.88	3.1	2.9×10^{-2}
		cell cycle	16	5.49	2.9	3.1×10^{-2}

Supplementary Figure 3. Gene ontology terms enriched among mRNAs with changed translational efficiency.
Enrichment for Gene Ontology terms (biological processes) was caculated using Panther database with two-tailed Fisher's exact test *p*-value adjusted for multiple comparisons with Benjamini-Hochberg procedure.

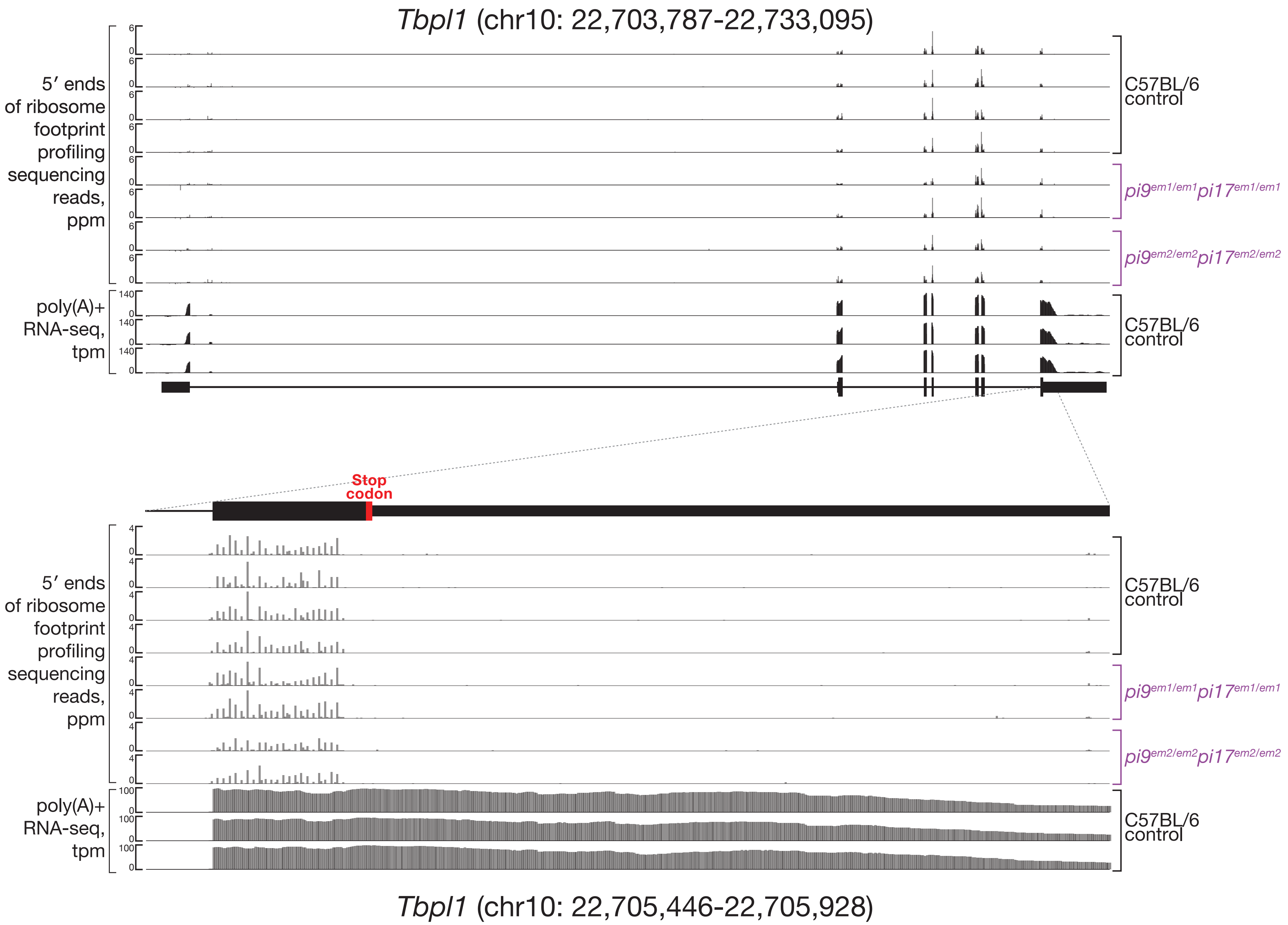

Supplementary Figure 4. RFP-seq and RNA-seq data for ***Tbpl1*** mRNA.
Individual biological replicates for C57BL/6 controls (*n* = 4 for RFP-seq, *n* = 3 for RNA-seq) and $pi9^{-/-}pi17^{-/-}$ (*n* = 4 for RFP-seq) are shown.

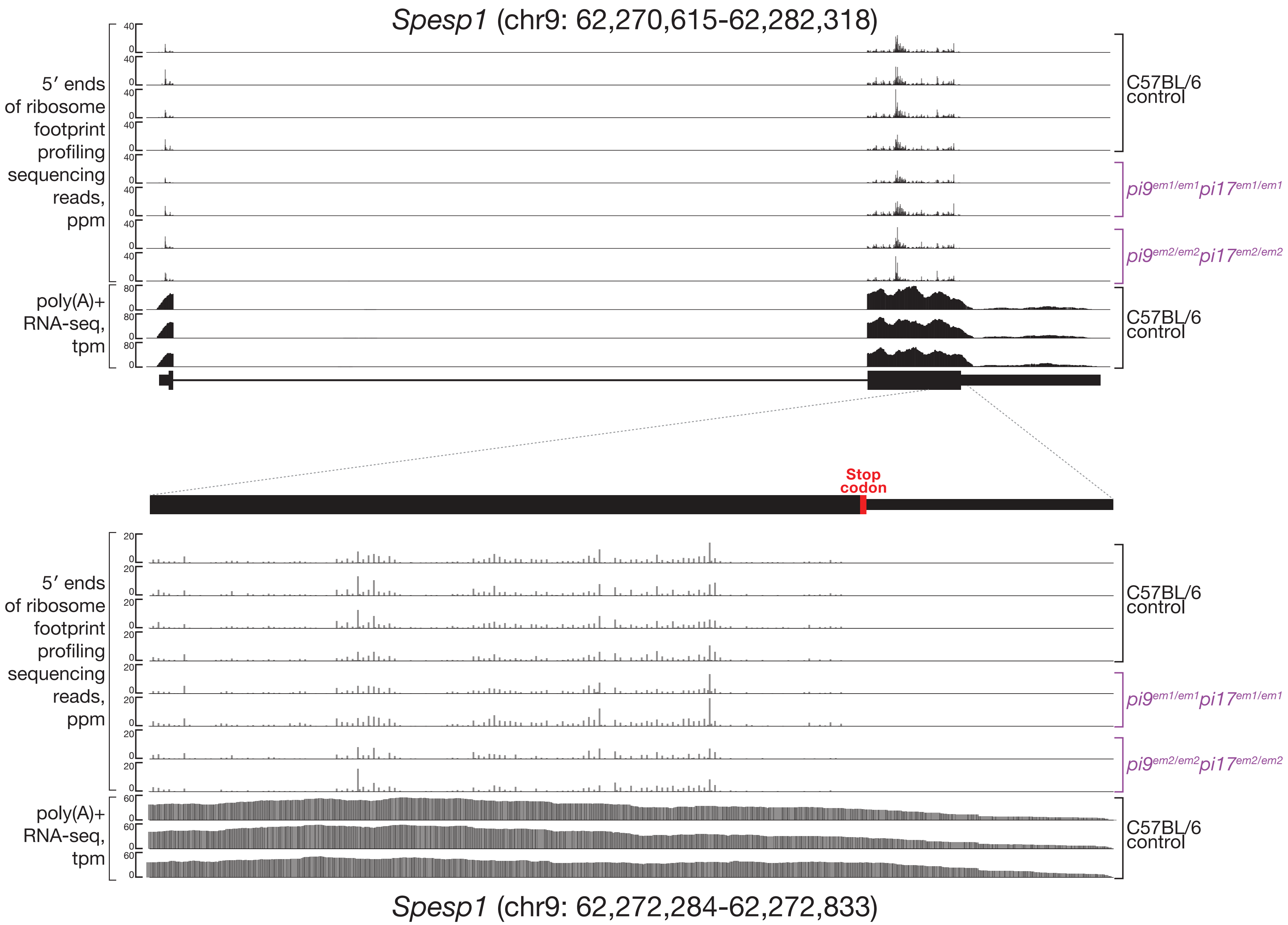

Supplementary Figure 5. RFP-seq and RNA-seq data for ***Spesp1*** mRNA.
Individual biological replicates for C57BL/6 controls (n = 4 for RFP-seq, n = 3 for RNA-seq) and $pi9^{-/-}pi17^{-/-}$ (n = 4 for RFP-seq) are shown.

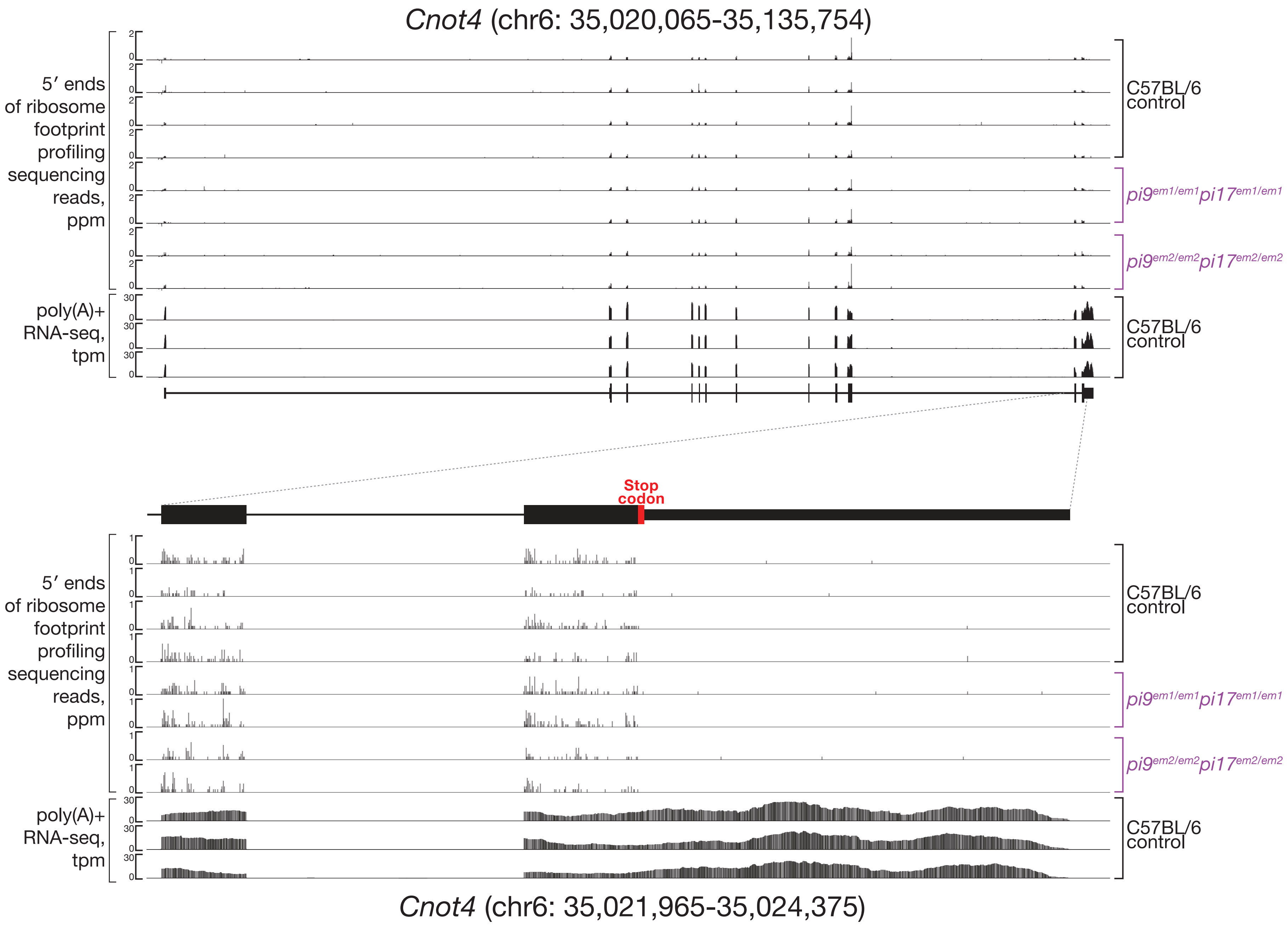

Supplementary Figure 6. RFP-seq and RNA-seq data for ***Cnot4*** mRNA.
Individual biological replicates for C57BL/6 controls (*n* = 4 for RFP-seq, *n* = 3 for RNA-seq) and *pi9*$^{-/-}$*pi17*$^{-/-}$ (*n* = 4 for RFP-seq) are shown.

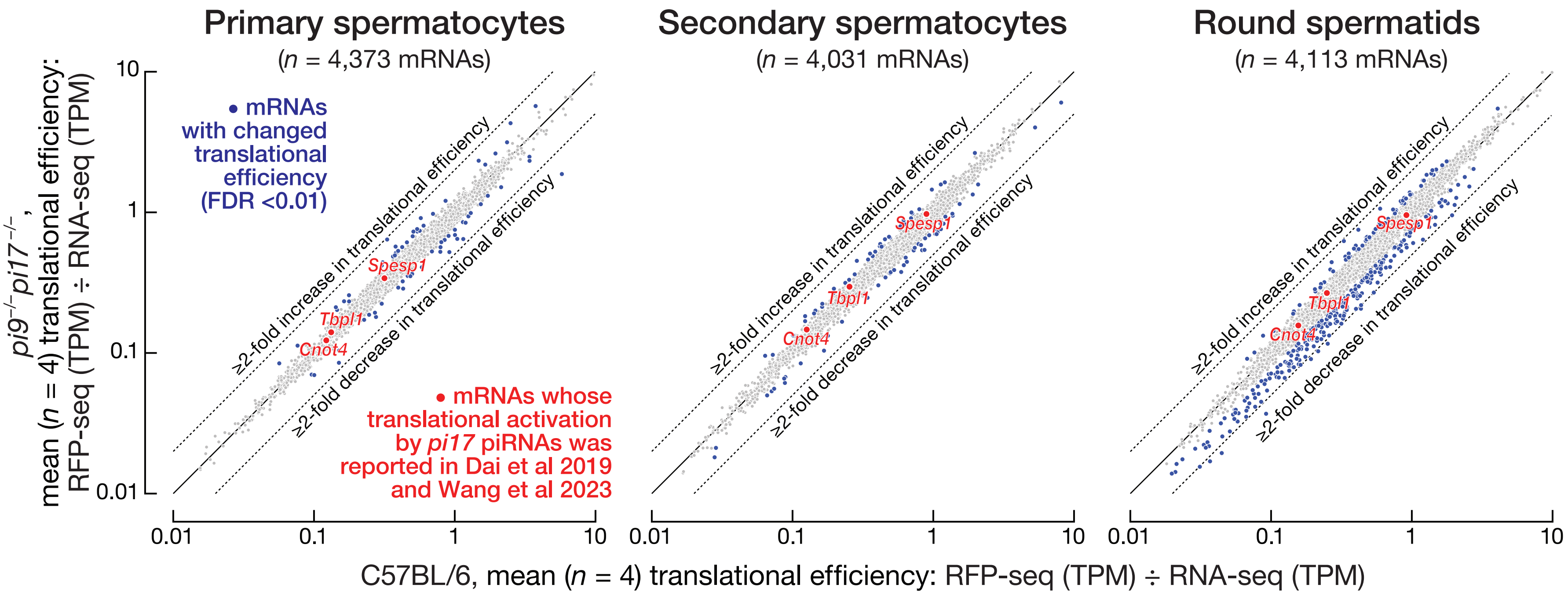

Supplementary Figure 7. Change in mRNA translational efficiency in pachytene piRNA mutants. Change in mean translational efficiency of mRNAs in FACS-purified *pi9*$^{-/-}$*pi17*$^{-/-}$ (*n* = 4) vs C57BL/6 (*n* = 4) primary spermatocytes, secondary spermatocytes, and round spermatids. Data are for all mRNAs with ≥10 TPM ribosome occupancy in each cell type. mRNAs with significantly changedribosome occupancy (FDR<0.01) are shown in blue and were identified using Benjamini-Hochberg-corrected *p*-values for two-tailed Wald test calculated by DESeq2. mRNAs whose translational activation by *pi17* piRNAs was reported in Dai et al 2019 and Wang et al 2023 are shown in red.

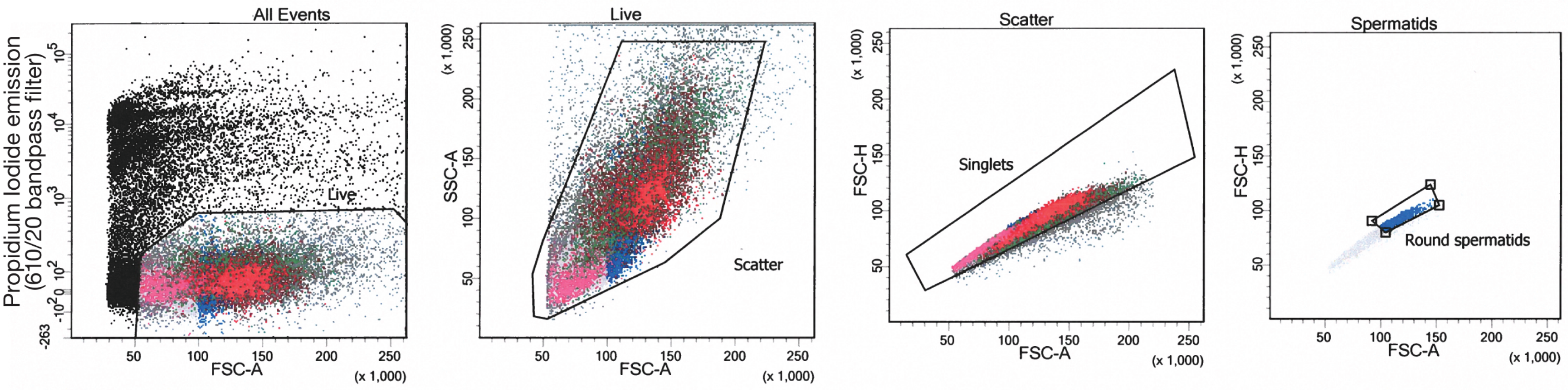

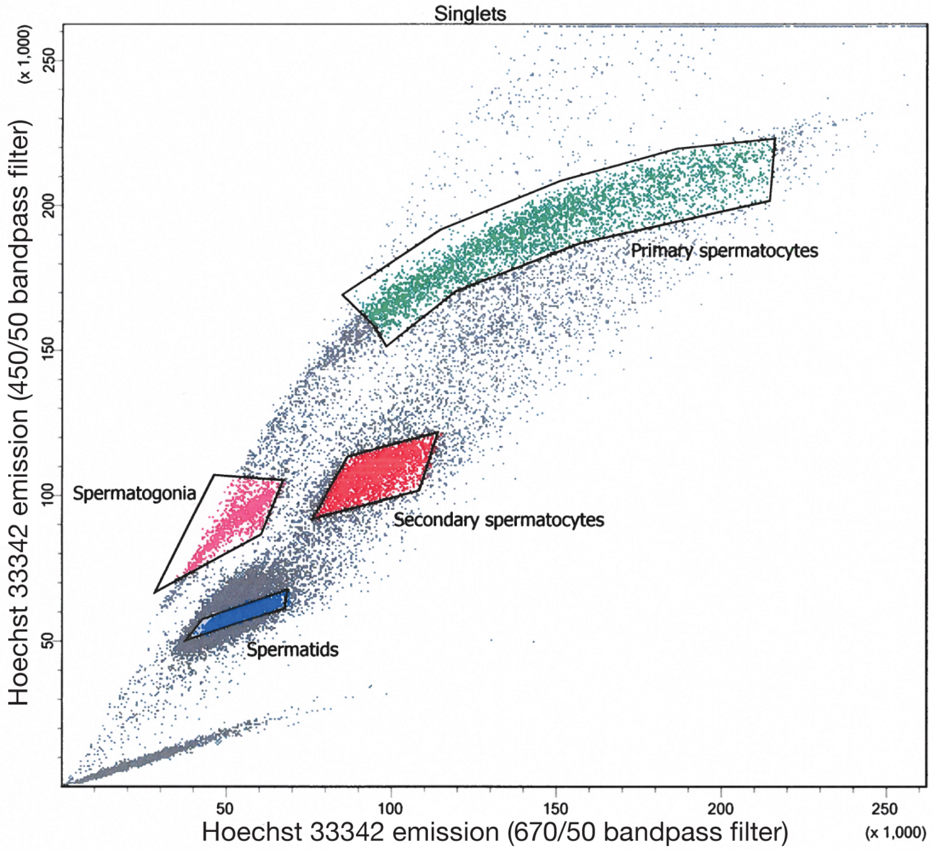

Population	#Events	%Parent	%Total
All Events	70,076	####	100.0
Live	53,149	75.8	75.8
Scatter	51,149	96.2	73.0
Singlets	50,032	97.8	71.4
Spermatogonia	1,264	2.5	1.8
Secondary spermatocytes	3,178	6.4	4.5
Spermatids	21,769	43.5	31.1
Round spermatids	5,086	23.4	7.3
Primary spermatocytes	4,163	8.3	5.9

Supplementary Figure 8. FACS gating strategy to purify mouse primary germ cells
