# Supplementary material for: Cleavage of mRNAs by a minority of pachytene piRNAs improves sperm fitness
Source: Nature. 2026 Feb 4;652(8109):508–16. doi: 10.1038/s41586-026-10102-9 (PMC13061629; doi:10.1038/s41586-026-10102-9)
Supplement: Supplementary file 4 — Mouse strains used in this study. Known or proposed molecular function for genes for which abundance changes in pi9−/− or pi17−/− primary spermatocytes versus C57BL/6. Oligonucleotides used in this study. Number of primary spermatocytes and amount of spike-in mix used to prepare small RNA-seq libraries. [file 41586_2026_10102_MOESM4_ESM.pdf]

Supplementary Table 1. Mouse strains used in this study.

Strain	Two guide RNAs sequences	Deletion coordinates (mm10)	Genotyping mutant allele		Genotyping wild-type allele	
			Primers	Amplicon size	Primers	Amplicon size
$pi2^{-/-}$<br>($pi2^{em1Pdz/ em1Pdz}$)<br>MGI 7545672	GCT TGA TCG<br>TCA GGG ACT AA<br><br>TCA GAG GCT<br>AAG TCC CAT TA	chr2:92539403–92542146	CCA CCT CCA GCT CTT<br>CCT CT<br><br>TTA GCT GCC TCA AGA<br>GTG GC	Mut 732 bp	CCC TTG ATC ATA CCC<br>ACC TCC<br><br>TGT CAA CAA ACC CCC<br>AGG AC	501 bp
$pi6^{-/-}$<br>($pi6^{em1Pdz/ em1Pdz}$)<br>reported in Wu et al.,<br>*Nat Genet* 2020;<br>MGI 6441985	GAC TGC CTA<br>CTC CAA GAT AG<br><br>CAC ACA AGT<br>GCC CAA CGA AA	chr6:127796350<br>-127796474	ATC CTC CCA GAT GGC<br>TCT GT<br><br>TGC CCA CTT TAC TGA<br>GGC TG	WT 984 bp<br>Mut 767 bp	GGC CAC TGG CAG TTA<br>GTT CT<br><br>TGC CCA CTT TAC TGA<br>GGC TG	396 bp
$pi6^{-/-}$<br>($pi6^{em2Pdz/ em2Pdz}$)<br>reported in Wu et al.,<br>*Nat Genet* 2020;<br>MGI 6441998	ACG GTG GGT<br>TCT ATC CAA TG<br><br>GGA TAG AGT<br>AAG TGA GAA GC	chr6:127796350<br>-127796474	ATC CTC CCA GAT GGC<br>TCT GT<br><br>TGC CCA CTT TAC TGA<br>GGC TG	WT 984 bp<br>Mut 859 bp	GGC CAC TGG CAG TTA<br>GTT CT<br><br>TGC CCA CTT TAC TGA<br>GGC TG	396 bp
$pi7^{-/-}$<br>($pi7^{em1Pdz/ em1Pdz}$)<br>MGI 7545673	CCG GGG CCT<br>GCA AAG AAG AA<br><br>GAC CAC CCT<br>GAA ACC TGT AA	chr7:73816369-73816663	CAT GT CGT TGC TGG<br>GCA AAA<br><br>GTG GAC CTG TTG CAG<br>GAA CT	WT 983 bp<br>Mut 664 bp	CCC TTT GCC TAG GAC<br>TGT GG<br><br>CAT GTC GTT GCTG GGC<br>AAA A	488 bp
$pi7^{-/-}$<br>($pi7^{em2Pdz/ em2Pdz}$)<br>MGI 7767968	ACA GGG ATA<br>ATA GCT ATC CC<br><br>CAC TAG GAT<br>TCC CGT ATC AG	chr7:73816243-73816716	CAT GT CGT TGC TGG<br>GCA AAA<br><br>GTG GAC CTG TTG CAG<br>GAA CT	WT 983 bp<br>Mut 510 bp	CCC TTT GCC TAG GAC<br>TGT GG<br><br>CAT GTC GTT GCTG GGC<br>AAA A	488 bp
$pi9^{-/-}$<br>($pi9^{em1Pdz/ em1Pdz}$)<br>MGI 7545674	GGC CTG CAG<br>CAT GCT CTT GC<br><br>GTT TAG GGT<br>TTG GGT AAG TT	chr9:67733702–67734069	AGA TCC AGA GGC AGG<br>CTT TT<br><br>TGC CAG CT CTC TTG<br>TCA GAA	WT 774 bp<br>Mut 393 bp	CGT GGA CAA CAG GGA<br>CAC TA<br><br>CCA CCC CAA ATG CCA<br>TGA AG	307 bp

pi9$^{-/-}$ (*pi9*$^{em2Pdz/ em2Pdz}$) MGI 7778314	AGA GTA CGA GGC TAT ACG GA<br><br>AAT AAT CCC ACG GAC TCA CC	chr9:67733639-67734194	AGA TCC AGA GGC AGG CTT TT<br><br>TGC CAG CT CTC TTG TCA GAA	WT 774 bp<br>Mut 219 bp	CGT GGA CAA CAG GGA CAC TA<br><br>CCA CCC CAA ATG CCA TGA AG	307 bp
pi17$^{-/-}$ (*pi17*$^{em1Pdz/ em1Pdz}$) reported in Wu et al., *Nat Genet* 2020; MGI 6441981	GTC CCT TCA CAC GGC CGT TTA<br><br>GCT CTG TCT GAC AAC GGG AC	chr17:27324887–27325439	CGC AGC CCA TCC ATT TCT TG<br><br>GAC TAG CGC CAG TTT CCA CT	WT 1000 bp<br>Mut 448 bp	AGG TCT GCA CGT AGT CTC CT<br><br>GGG TGT GGC CAC ATG TAT CA	368 bp
pi17$^{-/-}$ (*pi17*$^{em2Pdz/ em2Pdz}$) MGI 6441982	ACC GCT GCG CGC CGT GGG AC<br><br>CTG GGA ATC CGG GGT AGC GG	chr17:27324971-27325488	CGC AGC CCA TCC ATT TCT TG<br><br>GAC TAG CGC CAG TTT CCA CT	WT 1000 bp<br>Mut 483 bp	AGG TCT GCA CGT AGT CTC CT<br><br>GGG TGT GGC CAC ATG TAT CA	368 bp

Supplementary Table 4a. Molecular function of genes whose abundance changes significantly in *pi9*$^{-/-}$ primary spermatocytes (FDR<0.01).

Gene (*-pi9 target)	Fold change *pi9*$^{-/-}$/ *C57BL/6*	Molecular function	References
Aen*	2.4	ssDNA and dsDNA exonuclease, induces apoptosis	(Kawase et al., 2008)
Zbtb26*	2.1	Transcription factor	(Mance et al., 2024)
Champ1*	1.8	Regulator of homologous recombination in DNA damage response	(Li et al., 2022)
Brca2*	1.7	Directs RAD51 to ssDNA during DNA damage response	(Holloman, 2011)
Mrpl27	1.6	Nuclear encoded mitochondrial ribosomal protein	(Gruschke et al., 2010)
Gzf1*	1.5	Transcription factor; implicated in regulation of cell proliferation	(Morinaga et al., 2005; Dambara et al., 2007)
Idh1	0.4	Isocitrate dehydrogenase (cytoplasmic)	(Pirozzi and Yan, 2021)

Supplementary Table 4b. Molecular function of genes whose abundance changes significantly in *pi17*$^{-/-}$ primary spermatocytes (FDR<0.01).

Gene (*-pi17 target)	Fold change *pi17*$^{-/-}$/ *C57BL/6*	Molecular function	References
Slc41a1*	4.4	Magnesium transporter	(Schäffers et al., 2018; Ilenwabor et al., 2022)
Paqr8*	2.1	Member of progestin and adipoQ receptor (PAQR) protein family; required for tumor survival	(Chen et al., 2023; Pilon and Ruiz, 2023)
Urgcp*	2.0	Upregulator Of Cell Proliferation; contains very large inducible GTPase (VLIG)-type guanine nucleotide-binding domain; implicated in regulation of cell proliferation	(Xie et al., 2012; Xing et al., 2015)
Ywhaz*	2.0	Member of 14-3-3 protein family that regulate signaling pathways; implicated in control of cell proliferation	(Li et al., 2010; Nishimura et al., 2013)
Zfp473*	1.9	Zinc-finger protein	
Acsl3*	1.8	Long-chain acyl-coenzyme A synthase; implicated in regulation of cell proliferation	(Sebastiano et al., 2020; Saliakoura et al., 2020)
Gm11635	1.7	121-aa protein with 25% identity to *Pongo pygmaeus* BRCA1 amino acid residues 899–1022	
Zdhhc16*	1.7	Zinc-finger containing palmitoyltransferase; implicated in regulation of cell proliferation	(Sun et al., 2022)
Chp1*	1.5	Regulator of endoplasmic reticulum glycerolipid synthesis	(Zhu et al., 2019)
Tktl2	1.5	Member of transketolase protein family	(Deshpande et al., 2019)
Cox7a2l*	1.4	Regulator of mitochondrial respirasome biogenesis; implicated in regulation of cell proliferation	(Lobo-Jarne et al., 2018; Ikeda et al., 2019)

References from Supplementary Table 4.

Chen, Saisai, et al. (2023), ‘PAQR8 promotes breast cancer recurrence and confers resistance to multiple therapies’, *Breast Cancer Research*, 25 (1), 1.

Dambara, A, et al. (2007), ‘Nucleolin modulates the subcellular localization of GDNF-inducible zinc finger protein 1 and its roles in transcription and cell proliferation.’, *Exp Cell Res*, 313 (17), 3755-66.

Deshpande, GP, HG Patterton, and M Faadiel Essop (2019), ‘The human transketolase-like proteins TKTL1 and TKTL2 are bona fide transketolases.’, *BMC Struct Biol*, 19 (1), 2.

Gruschke, S, et al. (2010), ‘Proteins at the polypeptide tunnel exit of the yeast mitochondrial ribosome.’, *J Biol Chem*, 285 (25), 19022-28.

Holloman, William K (2011), ‘Unraveling the mechanism of BRCA2 in homologous recombination’, *Nature structural & molecular biology*, 18 (7), 748-54.

Ikeda, K, et al. (2019), ‘Mitochondrial supercomplex assembly promotes breast and endometrial tumorigenesis by metabolic alterations and enhanced hypoxia tolerance.’, *Nat Commun*, 10 (1), 4108.

Ilenwabor, BP, et al. (2022), ‘SLC41A1 knockout mice display normal magnesium homeostasis.’, *Am J Physiol Renal Physiol*, 323 (5), F553-63.

Kawase, Tatsuya, et al. (2008), ‘p53 target gene AEN is a nuclear exonuclease required for p53-dependent apoptosis’, *Oncogene*, 27 (27), 3797-810.

Li, Feng, et al. (2022), ‘CHAMP1 binds to REV7/FANCV and promotes homologous recombination repair’, *Cell reports*, 40 (9),

Li, Y, et al. (2010), ‘Amplification of LAPTM4B and YWHAZ contributes to chemotherapy resistance and recurrence of breast cancer.’, *Nat Med*, 16 (2), 214-18.

Lobo-Jarne, T, et al. (2018), ‘Human COX7A2L Regulates Complex III Biogenesis and Promotes Supercomplex Organization Remodeling without Affecting Mitochondrial Bioenergetics.’, *Cell Rep*, 25 (7), 1786-1799.e4.

Mance, L, et al. (2024), ‘Dynamic BTB-domain filaments promote clustering of ZBTB proteins.’, *Mol Cell*, 84 (13), 2490-2510.e9.

Morinaga, T, et al. (2005), ‘GDNF-inducible zinc finger protein 1 is a sequence-specific transcriptional repressor that binds to the HOXA10 gene regulatory region.’, *Nucleic Acids Res*, 33 (13), 4191-201.

Nishimura, Y, et al. (2013), ‘Overexpression of YWHAZ relates to tumor cell proliferation and malignant outcome of gastric carcinoma’, *British journal of cancer*, 108 (6), 1324-31.

Pilon, M and M Ruiz (2023), ‘PAQR proteins and the evolution of a superpower: Eating all kinds of fats: Animals rely on evolutionarily conserved membrane homeostasis proteins to compensate for dietary variation.’, *Bioessays*, 45 (9), e2300079.

Pirozzi, CJ and H Yan (2021), ‘The implications of IDH mutations for cancer development and therapy.’, *Nat Rev Clin Oncol*, 18 (10), 645-61.

Saliakoura, Maria, et al. (2020), ‘The ACSL3-LPIAT1 signaling drives prostaglandin synthesis in non-small cell lung cancer’, *Oncogene*, 39 (14), 2948-60.

Schäffers, OJM, et al. (2018), ‘The rise and fall of novel renal magnesium transporters.’, *Am J Physiol Renal Physiol*, 314 (6), F1027-33.

Sebastiano, M Rossi, C Pozzato, and M Saliakoura… (2020), ‘ACSL3–PAI-1 signaling axis mediates tumor-stroma cross-talk promoting pancreatic cancer progression’, *Science …*,

Sun, Y, et al. (2022), ‘S-palmitoylation of PCSK9 induces sorafenib resistance in liver cancer by activating the PI3K/AKT pathway.’, *Cell Rep*, 40 (7), 111194.

Xie, C, et al. (2012), ‘Upregulator of cell proliferation predicts poor prognosis in hepatocellular carcinoma and contributes to hepatocarcinogenesis by downregulating FOXO3a.’, *PLoS One*, 7 (7), e40607.

Xing, Sizhong, et al. (2015), ‘URG4/URGCP enhances the angiogenic capacity of human hepatocellular carcinoma cells in vitro via activation of the NF-κB signaling pathway’, *BMC cancer*, 15 1-12.

Zhu, XG, et al. (2019), ‘CHP1 Regulates Compartmentalized Glycerolipid Synthesis by Activating GPAT4.’, *Mol Cell*, 74 (1), 45-58.e7.

Supplementary Table 9. Sequences of oligonucleotides used in this study

	Name	Sequence (5′-to-3′)	Notes
Adapters, oligos, and primers for small RNA sequencing and RFP (Ribosome Footprint Profiling)	Equimolar mix of nine spike-in RNA oligonucleotides, only used for small RNA sequencing	/phos/UGCUAGUCUGUUAUCGACCUGACCUCAUAG	5′ monophosphorylated RNA
		/phos/UGCUAGUCUGUUCGAUACCUGACCUCAUAG	
		/phos/UGCUAGUCUGUUGUCACGAAGACCUCAUAG	
		/phos/UGCUAGUCUUAUCGACCUCCUCAUAG	
		/phos/UGCUAGUCUUCGAUACCUCCUCAUAG	
		/phos/UGCUAGUCUUGUCACGAACCUCAUAG	
		/phos/UGCUAGUUAUCGACCUUCAUAG	
		/phos/UGCUAGUUCGAUACCUUCAUAG	
		/phos/UGCUAGUUGUCACGAAUCAUAG	
	3′ DNA adapter	/rApp/NNNGTCNNNTAGNNNTGGAATTCTCGGGTGCCAAGG/ddC/	5′ adenylated, 3′ dideoxycytosine blocked DNA adapter
	Equimolar mix of two 5′ RNA adaptors	GUUCAGAGUUCUACAGUCCGACGAUCNNNCGANNNUACNNN	RNA
		GUUCAGAGUUCUACAGUCCGACGAUCNNNAUCNNNAGUNNN	
	RT primer	CCTTGGCACCCGAGAATTCCA	DNA
	Forward (P5) primer	AATGATACGGCGACCACCGAGATCTACACGTTC AGAGTTCTACAGTCCGA	DNA primers for final amplification of library, XXXXXX represents 6-nt sequencing barcode
	Reverse (P7) primer	CAAGCAGAAGACGGCATACGAGATXXXXXX<br>GTGACTGGAGTTCCTTGGCACCCGAGAATTCCA	
Three sets of unique molecular identifier (UMI) containing RNAseq adapters	Adapter set 1	/phos/CCCNNNNNNAGATCGGAAGAGCACACGTCT	Two adapters in each set are first annealed to each other in three separate tubes, then the three annealed sets are equimolarly mixed to a final total concentration of 3.3 μM each duplex
		ACACTCTTTCCCTACACGACGCTCTTCCGATCTNNNNNNGGGT	
	Adapter set 2	/phos/GATNNNNNNAGATCGGAAGAGCACACGTCT	
		ACACTCTTTCCCTACACGACGCTCTTCCGATCTNNNNNNATCT	
	Adapter set 3	/phos/TGANNNNNNAGATCGGAAGAGCACACGTCT	
		ACACTCTTTCCCTACACGACGCTCTTCCGATCTNNNNNNTCAT	
Adapters and primers for cloning and sequencing long 5′ mono-phosphorylated RNAs	Equimolar mix of two 5′ RNA adaptors	GUUCAGAGUUCUACAGUCCGACGAUCNNNCGANNNUACNNN	RNA
		GUUCAGAGUUCUACAGUCCGACGAUCNNNAUCNNNAGUNNN	
	RT primer	GCACCCGAGAATTCCANNNNNNNNN	DNA
	PCR 1 forward primer	CTACACGTTCAGAGTTCTACAGTCCGA	DNA primers for the first PCR amplification

	PCR 1 reverse primer	GCCTTGGCACCCGAGAATTCCA	
	Forward (P5) primer	AATGATACGGCGACCACCGAGATCTACACGTTC AGAGTTCTACAGTCCGA	DNA primers for the second PCR amplification
	Reverse (P7) primer	CAAGCAGAAGACGGCATACGAGATXXXXXX GTGACTGGAGTTCCTTGGCACCCGAGAATTCCA	

Supplementary Table 10. Number of Primary Spermatocytes and Amount of Spike-In Mix Used to Prepare Small RNA Sequencing Libraries.

Genotype		Trial	Cell number	Amount of spike-in, attomol
C57BL/6 data are from Gainetdinov et al, *Nature* 2023	SRR21528503	Rep1	31400	370
	SRR21528502	Rep2	68900	4000
	SRR21528501	Rep3	47200	3000
	SRR21528500	Rep4	89100	4000
	SRR21528499	Rep5	48900	3000
	SRR21528498	Rep6	60300	4000
	SRR21528497	Rep7	63100	4000
	SRR21528495	Rep8	99700	4000
	SRR21528494	Rep9	112500	4000
	SRR21528493	Rep10	116400	4000
	SRR21528492	Rep11	137000	4000
	SRR21528491	Rep12	112000	4000
pi9$^{-/-}$		Rep1	41,600	3,000
		Rep2	47,100	3,000
		Rep3	45,600	3,000
		Rep4	59,600	4,000
		Rep5	71,200	4,000
		Rep6	95,800	4,000
		Rep7	56,000	4,000
pi17$^{-/-}$		Rep1	59,900	4,000
		Rep2	36,460	4,000
		Rep3	76,570	4,000
		Rep4	54,000	4,000
		Rep5	153,300	4,000
		Rep6	91,300	4,000
pi9$^{-/-}$*pi17*$^{-/-}$		Rep1	40,000	4,000
		Rep2	36,000	4,000
		Rep3	33,500	4,000
pi6$^{-/-}$		Rep1	45,400	4,000
